# Contribution of DEAD-Box RNA Helicase 21 to the Nucleolar Localization of Porcine Circovirus Type 4 Capsid Protein

**DOI:** 10.3389/fmicb.2022.802740

**Published:** 2022-02-25

**Authors:** Jianwei Zhou, Yuexia Wang, Yonghui Qiu, Yongxia Wang, Xiaoyu Yang, Changzhe Liu, Yongyan Shi, Xufei Feng, Lei Hou, Jue Liu

**Affiliations:** ^1^College of Veterinary Medicine, Yangzhou University, Yangzhou, China; ^2^Jiangsu Co-innovation Center for Prevention and Control of Important Animal Infectious Diseases and Zoonoses, Yangzhou University, Yangzhou, China; ^3^Qingpu District Municipal Agriculture Commission, Shanghai, China; ^4^College of Animal Science, Fujian Agriculture and Forestry University, Fuzhou, China

**Keywords:** porcine circovirus type 4, capsid protein, nucleolar localization signal, DEAD-box RNA helicase 21, nucleolar localization

## Abstract

Porcine circovirus type 4 (PCV4) is a newly emerging pathogen which might be associated with diverse clinical signs, including respiratory and gastrointestinal distress, dermatitis, and various systemic inflammations. The host cellular proteins binding to PCV4 capsid (Cap) protein are still not clear. Herein, we found that the PCV4 Cap mediated translocation of DEAD-box RNA helicase 21 (DDX21) to the cytoplasm from the nucleolus and further verified that the nucleolar localization signal (NoLS) of the PCV4 Cap bound directly to the DDX21. The NoLS of PCV4 Cap and ^763^GSRSNRFQNK^772^ residues at the C-terminal domain (CTD) of DDX21 were required for this PCV4 Cap/DDX21 interaction. Further studies indicated that the PCV4 Cap NoLS exploited DDX21 to facilitate its nucleolar localization. In summary, our results firstly demonstrated that DDX21 binds directly to the NoLS of the PCV4 Cap thereby contributing to the nucleolar localization of the PCV4 Cap protein.

## Introduction

Porcine circoviruses (PCVs) belong to non-enveloped viruses containing single-stranded circular DNA genomes (∼1.7–2.0 kb) within genus *Circovirus* in the family *Circoviridae* (Breitbart et al., 2017). Four genotypes of circoviruses have been detected in pigs (Zhang et al., 2019; Oh and Chae, 2020; Opriessnig et al., 2020). PCV1 is non-pathogenic, while PCV2 is the main pathogen of porcine circovirus-associated diseases (PCVAD) (Tischer et al., 1982; Allan et al., 1998). PCV3 was newly discovered in 2016 (Phan et al., 2016; Palinski et al., 2017). PCV4, a novel PCV, which was first reported in Hunan province, China in 2019, was related to clinical symptoms, such as respiratory distress and porcine dermatitis and nephropathy syndrome (PDNS) (Zhang et al., 2019). Since then, PCV4 was detected in other pig-rearing provinces in China as well (Sun et al., 2020; Tian et al., 2020; Chen et al., 2021; Ha et al., 2021), indicating that PCV4 is probably prevalent in Chinese swine farms. Likewise, PCV4 was found in South Korea but not detected in Italy and Spain (Franzo et al., 2020; Nguyen et al., 2021).

Circoviruses depend on the host cellular replication machinery for viral genome synthesis owing to shortage of autonomous DNA polymerases (Heath et al., 2006). As for all PCVs, the conserved N-terminus of Cap protein comprise a nuclear localization signal (NLS) (Liu et al., 2001; Shuai et al., 2008; Mou et al., 2019). The amino acids of PCV4 Cap are extremely different from those of PCV1, PCV2, and PCV3, but their motifs are highly similar within the NLSs in spite of different PCV genotypes (Liu et al., 2001; Shuai et al., 2008; Mou et al., 2019). The viral proteins bearing NLS are essential for virus replication, protein translation, and progression and division of cell cycle (Wurm et al., 2001; Hiscox, 2002; Salvetti and Greco, 2014). Thus, mapping the cellular host proteins binding to PCV4 Cap bearing a NoLS will help understand the replication and pathogenesis of PCV4 infection.

DEAD-box RNA helicases are a myriad of multifunctional enzymes that control multiple processes of RNA metabolism, such as transcription, processing, and modification (Linder and Jankowsky, 2011; Zhang et al., 2011; Hao et al., 2019; Cargill et al., 2021; Ullah et al., 2021). Sometimes, these proteins function in microRNA processing, RNA nuclear transport and decoy as well (Diot et al., 2016). DEAD-box RNA helicase 21 (DDX21) was considered as a plenteous nucleolar protein that connects with ribosomal RNA (rRNA) and small nucleolar RNAs (snoRNAs) to facilitate RNA metabolism (Flores-Rozas and Hurwitz, 1993; Valdez et al., 1996; Henning et al., 2003; Holmstrom et al., 2008; Calo et al., 2015). DDX21 is composed of a largely conserved central helicase domain carrying the DEXD sequence and flanking N-terminal and C-terminal domains that are highly variable and proposed to bind to multifarious host proteins and/or RNAs (Fuller-Pace, 2006). To date, some researches have showed that DDX21 is important for governing RNA virus replication. For example, the cellular DDX21 restrains replication of influenza A virus via interaction with viral PB1 protein, repressing polymerase activity and causing decreased virus progeny production (Chen et al., 2014). During early phase of dengue virus infection, DDX21 was reported to relocate to the cytoplasm from the nucleolus for the end of suppressing virus replication (Dong et al., 2016). Likewise, DDX21 was found to modulate Borna disease virus replication by regulating polycistronic mRNA translation (Watanabe et al., 2009). Furthermore, DDX21 also regulates host immune responses. For instance, DDX1, DDX21, and DHX36 can form a complex to govern cellular immune responses modulated by interferon, and deprivation of any component of the compound would repress host responses (Fullam and Schroder, 2013). In addition, caspase-dependent cleavage of DDX21 disrupts cellular anti-virus innate immunity (Wu et al., 2021). Based on these studies, DDX21 can modulate host cellular responses to viruses and plays crucial roles in virus replication. However, these studies have just explored RNA viruses, and the association between DDX21 and DNA virus has only been investigated once (Hao et al., 2019). Thus, it is necessary to ascertain whether DDX21 regulates porcine circovirus lifecycle and the underlying mechanism.

In this study, we found that DDX21 traffics to the cytoplasm from the nucleolus induced by the PCV4 Cap overexpression and the NoLS of the PCV4 Cap and ^763^GSRSNRFQNK^772^ residues at the CTD of DDX21 are essential for the PCV4 Cap/DDX21 interaction. In addition, the PCV4 Cap NoLS deployed DDX21 to promote its nucleolar localization. To sum up, these results firstly uncovered that DDX21 directly interacts with the PCV4 Cap NoLS and this interaction is critical for nucleolar retention of the PCV4 Cap protein.

## Results

### Porcine Circovirus Type 4 Cap Expression Resulted in the Redistribution of DDX21

**FIGURE 1:**
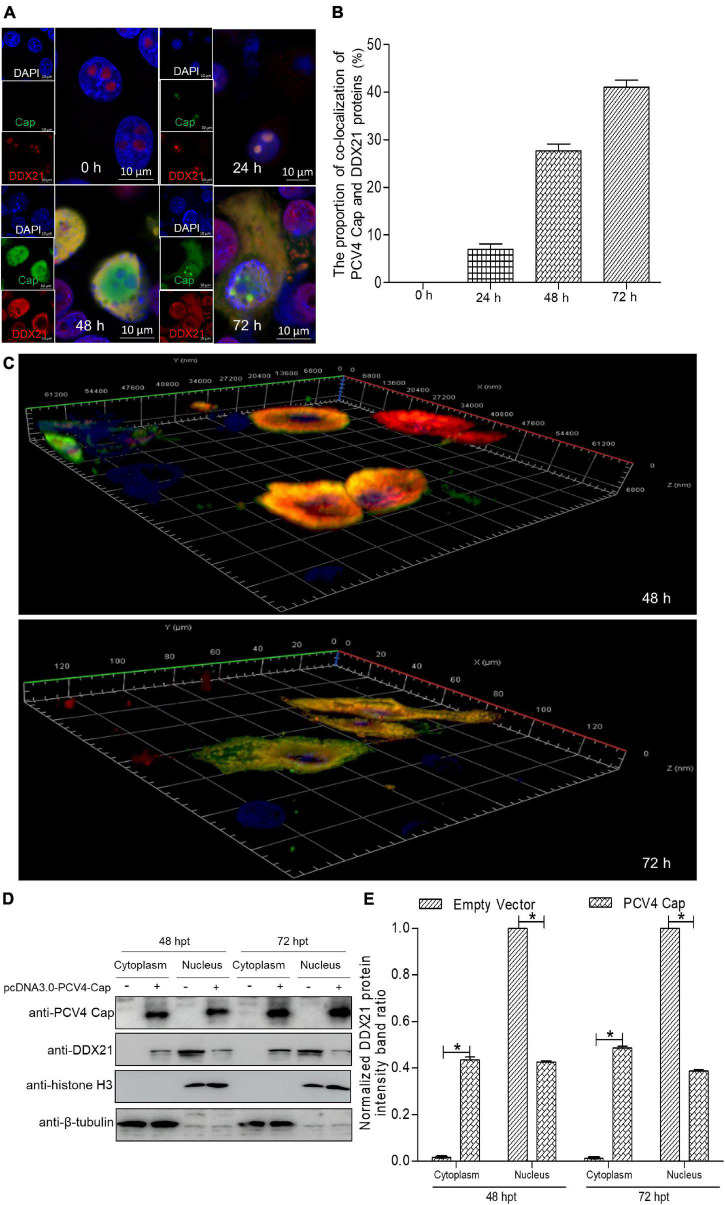
DDX21 relocates from the nucleolus to the cytoplasm induced by PCV4 Cap overexpression. **(A)** Immunofluorescence analysis of DDX21 localization during PCV4 Cap expression. PK-15 cells were transfected 4.0 μg of pcDNA3.0-PCV4-Cap plasmid. The cells were fixed at 24, 48, and 72 h post-transfection (hpt) and incubated with the antibodies corresponding PCV4 Cap, and DDX21 followed by the fluorescein isothiocyanate (FITC)-conjugated goat anti-mouse IgG (green) and Alexa Fluor-546 conjugated donkey anti-rabbit IgG (red) secondary antibodies. Nuclei were stained with DAPI (blue) and then observed under a confocal microscope. Scale bar, 10μm. **(B)** The proportion of co-localization of PCV4 Cap and DDX21 proteins was analyzed using ImageJ software at 24, 48, and 72 hpt. **(C)** The three-layer dimensional confocal stack analysis was performed to verify the co-localization of PCV4 Cap and DDX21 proteins at 48 and 72 hpt. **(D)** The cell nuclear and cytoplasmic fractions were extracted after PK-15 cells transfected 4.0 μg of pcDNA3.0-PCV4-Cap plasmid. At 48 and 72 hpt, the protein samples were prepared and analyzed by western blotting using antibodies against PCV4 Cap and DDX21. Histone H3 and β-tubulin served as fractionation quality controls. **(E)** The DDX21 protein band intensity was analyzed using ImageJ software at 48, 72 hpt. Data are presented as means ± SD of three independent experiments. *p < 0.05.

Although DDX21 is known to be a nucleolar protein within the DEAD-box RNA helicase family, its localization can be altered upon certain types of stimulation (Dong et al., 2016). For the sake of demonstrating whether the DDX21’s localization was altered during PCV4 Cap overexpression, we determined distribution of DDX21 in PK-15 cells by confocal microscopy. PK-15 cells were transfected with pcDNA3.0-PCV4-Cap, and fixed at 24, 48, and 72 h post-transfection (hpt). The localization of PCV4 Cap and DDX21 were detected by confocal microscopy. The results demonstrated that localization of PCV4 Cap to endogenous DDX21 (about 7.0% co-localization within cells) was observed in the nucleolus at 24 hpt and DDX21 was mainly resided in the nucleolus in mock-transfected cells (Figures 1A,B). Subsequently, the subcellular localization of endogenous DDX21 altered at 48 hpt, and partial DDX21 was relocated from the nucleolus to the nucleoplasm and overlapped with PCV4 Cap (about 27.7% co-localization within cells). The nucleolar localization of pcDNA3.0-PCV4-Cap and DDX21 in transfected PK-15 cells disappeared at 72 hpt, and they colocalized in the cytoplasm (about 41.0% co-localization within cells) (Figures 1A,B). The three-layer dimensional confocal stack analysis was performed to further verify the co-localization of PCV4 Cap and DDX21 proteins at 48 and 72 hpt (Figure 1C). A cytoplasmic-nuclear fractionation assay was further performed to evaluate the roles of PCV4 Cap-induced translocation. PK-15 cells were transfected with pcDNA3.0-PCV4-Cap and empty vector, and the subcellular fractions were isolated at 48 and 72 hpt. DDX21 was predominantly present in the cytoplasmic fraction after pcDNA3.0-PCV4-Cap transfection compared with cells transfected with empty vector, and cytoplasmic β-tubulin and nuclear histone H3 served as indicators of the subcellular fractionation (Figures 1D,E). Our results indicated that DDX21 relocated from the nucleolus to the cytoplasm in PCV4 Cap-transfected PK-cells during late phase.

### Porcine Circovirus Type 4 Cap Binds Directly to DDX21

**FIGURE 2:**
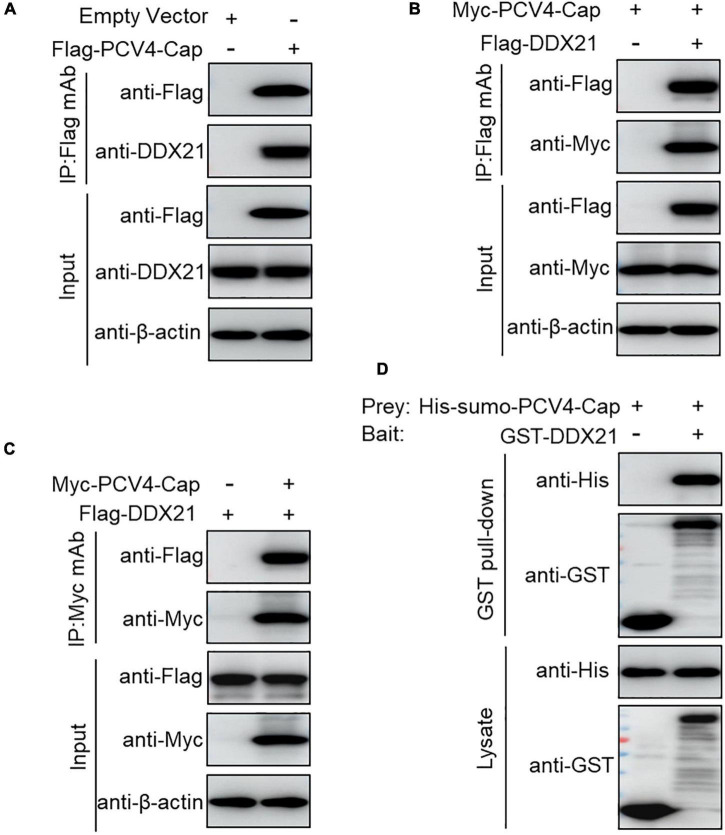
PCV4 Cap binds directly to DDX21. **(A)** PK-15 cells were transfected with empty vector or Flag-PCV4-Cap plasmid for 48 h. **(B,C)** The cell lysate extracts were immunoprecipitated with Flag beads **(A,B)** or anti-Myc purified IgG **(C)**. **(D)** The His-sumo-PCV4 Cap mixed with the GST, GST-DDX21 proteins were GST pulled-down and then analyzed by western blotting using corresponding antibodies.

To further explore the PCV4 Cap-induced DDX21 redistribution, Flag-PCV4-Cap-transfected PK-15 cell lysates were immunoprecipitated with anti-Flag beads and probed for DDX21 protein with anti-DDX21 mAb, showing that PCV4 Cap bound to the endogenous DDX21 protein (Figure 2A). Consistently, Flag-DDX21 and Myc-PCV4-Cap expression plasmids were cotransfected into HEK293T cells, and reciprocal immunoprecipitation was conducted using Flag beads or purified anti-Myc monoclonal antibodies (mAbs). As shown in Figures 2B,C, DDX21 interacted physically with PCV4 Cap protein. Furthermore, glutathione S-transferase (GST) affinity isolation revealed a direct interaction between PCV4 Cap and DDX21 (Figure 2D). Overall, these results clearly indicated that DDX21 could bind directly to PCV4 Cap.

### The Nucleolar Localization Signal of Porcine Circovirus Type 4 Cap Is Crucial for Interaction With DDX21

**FIGURE 3:**
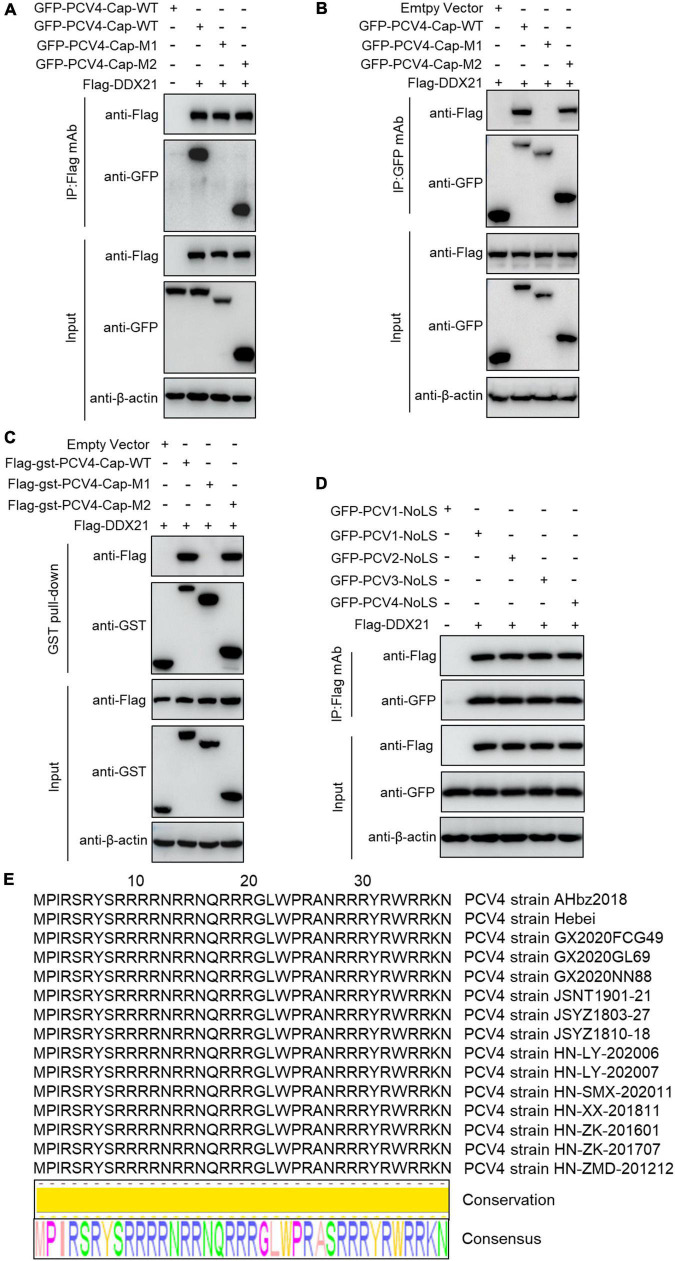
The N-terminal residues 1–37 of PCV4 Cap are essential for binding to DDX21. **(A–C)** HEK293T cells were co-transfected with plasmids containing full-length PCV4 Cap or truncated mutants fused with a GFP-, or Flag-GST tag, along with Flag-DDX21 plasmid for 48 h; the cell lysate extracts were immunoprecipitated with Flag beads **(A)** or anti-GFP purified IgG **(B)**, or pulled-down with glutathione S-transferase (GST) beads **(C)** and then detected by western blotting using the indicated antibodies. **(D)** The nucleolar localization signals (NoLSs) within capsid protein of PCV1, 2, 3, 4 were responsible for the binding to DDX21. HEK293T cells were cotransfected with plasmids encoding NoLSs of PCV1, 2, 3, 4, along with Flag-DDX21; cell lysates were subjected to immunoprecipitation and immunoblotting using the indicated antibodies. **(E)** Amino acid sequences alignment of the NoLSs from different PCV4 strains.

To map the domain (mainly NoLS) in PCV4 Cap essential for binding to DDX21, plasmids GFP-PCV4-Cap-WT, -M1, and -M2 or Flag-gst-PCV4-Cap-WT, -M1, and -M2 were, respectively cotransfected into HEK293T cells together with Flag-DDX21. Reciprocal Co-IP and GST pull-down assays indicated that amino acids (aa) 1–37 (M2) and the full-length PCV4 Cap (WT) could bind to DDX21, whereas aa 38–228 (M1) of PCV4 Cap were not able to interact with DDX21 (Figures 3A–C). These results indicated that the N-terminus 1–37 of PCV4 Cap are critical for Cap interaction with DDX21. Further Co-IP experiments demonstrated that the NoLSs within capsid protein of PCV1, 2, 3, 4 were required for interaction with DDX21 as well (Figure 3D), showing that the binding is evolutionarily conserved. Alignment of the Cap NoLS amino acid sequences from different PCV4 strains deposited in GenBank using Jalview software revealed that the NoLS of PCV4 Cap is wildly present and identical (Figure 3E).

### ^763^GSRSNRFQNK^772^ of DDX21 Is Crucial for Binding to Porcine Circovirus Type 4 Cap

**FIGURE 4:**
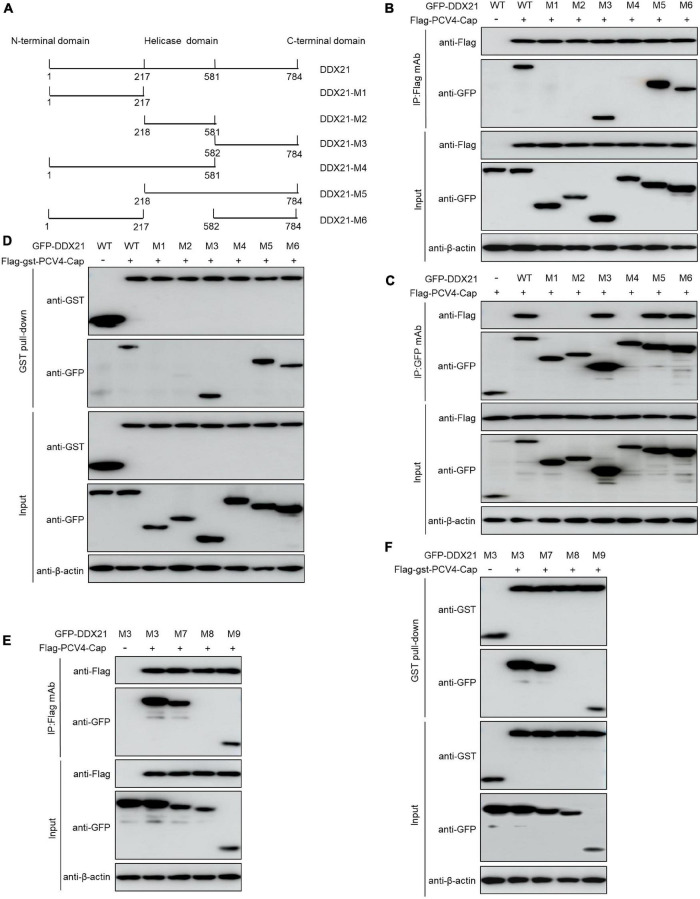
^763^GSRSNRFQNK^772^ of DDX21 is crucial for binding to PCV4 Cap. **(A)** Schematic representation of the NTD, Helicase D, and CTD of DDX21 and their truncation mutants used in this study. **(B–D)** The DDX21-CTD-(582–784 aa) interacted with PCV4 Cap. HEK293T cells were co-transfected with expression plasmids GFP- DDX21-WT or its serial GFP-DDX21 truncated mutants M1 to M6, together with Flag-PCV4-Cap or Flag-gst-PCV4-Cap plasmid. The cell lysate extracts were immunoprecipitated or GST pulled-down followed by western blotting using the indicated antibodies. **(E,F)** Identification the critical amino acids of DDX21-CTD essential for interaction with PCV4 Cap. HEK293T cells were co-transfected with DDX21 or DDX21 truncated mutants M7 to M9, along with Flag-PCV4-Cap or Flag-gst-PCV4-Cap plasmid, and the cell lysate extracts were immunoprecipitated or GST pulled-down followed by western blotting using the indicated antibodies.

DDX21 carries a N-terminal domain, a highly conserved central helicase domain, and a varied C-terminal domain (Fuller-Pace, 2006). To identify the domain required for binding of DDX21 to PCV4 Cap, plasmids GFP-DDX21-WT-(1–784 aa), GFP-DDX21-NTD-M1-(1–217 aa), GFP-DDX21-Helicase-M2-(218–581 aa), GFP-DDX21-CTD-M3-(582–784 aa), GFP-DDX21-NTD-Helicase-M4-(1–581 aa), GFP-DDX21-Helicase-CTD-M5-(218–784 aa), and GFP-DDX21-NTD-CTD-M6-(1–217 aa + 582–784 aa) were respectively, cotransfected into HEK293T cells along with Flag-PCV4-Cap or Flag-gst-PCV4-Cap. Reciprocal Co-IP and GST pull-down assays demonstrated that the constructs DDX21-CTD, DDX21-Helicase-CTD, and DDX21-NTD-CTD interacted with PCV4 Cap, whereas DDX21-NTD, DDX21-Helicase, and DDX21-NTD-Helicase did not (Figures 4A–D), indicating that the DDX21-CTD is required for binding to PCV4 Cap.

To characterize the key amino acid residues in the DDX21-CTD essential for binding to PCV4 Cap, we employed online tools (NucleOlar location sequence Detector^1^ and NLS Mapper^2^).^1,2^ A potential NoLS (^763^GSRSNRFQNK^772^) was predicted at the C-terminal of DDX21. Therefore, we cotransfected a series of mutants of GFP-DDX21-CTD, including GFP-DDX21-CTD-M7-(582–772 aa), GFP-DDX21-CTD-M8-(582–762 aa), and GFP-DDX21-CTD-M9-(763–772 aa) into HEK293T cells, and subjected to Co-IP and GST pull-down assays with Flag-PCV4-Cap or Flag-gst-PCV4-Cap. The results indicated that the mutants GFP-DDX21-CTD-M7-(582–772 aa) and GFP-DDX21-CTD-M9-(763–772 aa) bound with Flag-PCV4-Cap or Flag-gst-PCV4-Cap as well as the DDX21-CTD. However, GFP-DDX21-CTD-M8-(582–762 aa) mutant did not interact with PCV4 Cap (Figures 4E,F). In summary, these findings showed that ^763^GSRSNRFQNK^772^ of DDX21 is responsible for binding to PCV4 Cap.

### The DDX21 Is Indispensable for the Nucleolar Localization of Porcine Circovirus Type 4 Cap

**FIGURE 5:**
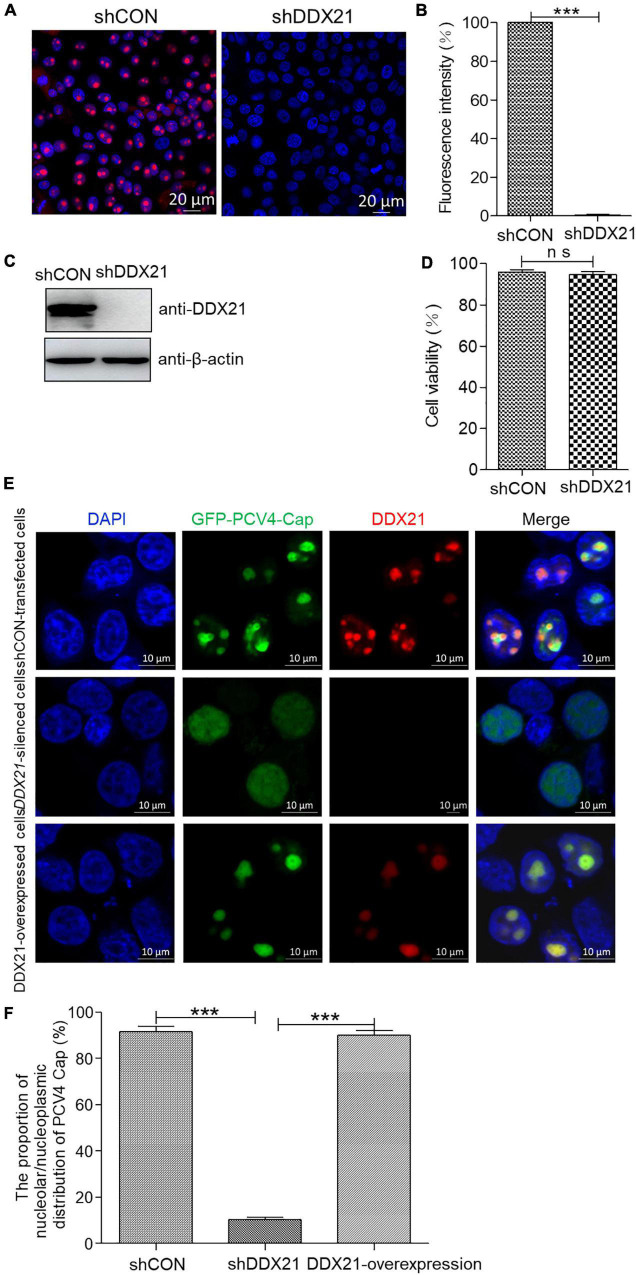
*DDX21* knockdown abrogated the nucleolar localization of PCV4 Cap. **(A)** Immunofluorescence analysis of DDX21 protein in shCON-transfected and *DDX21*-silenced PK-15 cells. The cells were incubated with the DDX21 antibody followed by the Alexa Fluor-546 conjugated donkey anti-rabbit IgG (red) secondary antibody. Nuclei were stained with DAPI (blue) and then observed under a confocal microscope. Scale bar, 20 μm. **(B)** The fluorescence intensity of DDX21 was analyzed using ImageJ software in shCON- and *DDX21*-silenced PK-15 cells. **(C)** The knockdown efficiency of DDX21 in shCON-transfected and *DDX21*-silenced PK-15 cells. The cell lysates were subjected to immunoblotting using the indicated antibodies. **(D)** The viability of PK-15 cells stably expressing a short hairpin RNA (shDDX21) was analyzed with a cell counting kit-8 (CCK-8) assay. **(E)** *DDX21*-knocked down and shCON-transfected and DDX21-overexpressed PK-15 cells were, respectively, transfected with GFP-PCV4-Cap for 24 h, and the cells were fixed and incubated with the DDX21 antibody and then subjected to confocal microscopic observation. Nuclei were stained with DAPI. Scale bar, 10 μm. **(F)** The fluorescence intensity of PCV4 Cap in the nucleolar and nucleoplasmic compartments was analyzed using ImageJ software. Data are presented as means ± SD of three independent experiments. ns, not significant; ***p < 0.001.

To assess the function of DDX21 in the nucleolar localization of PCV4 Cap, a short hairpin RNA (shRNA) specific for *DDX21* (shDDX21) and a non-targeting control shRNA (shCON) were transferred via lentivirus-mediated shRNA to produce cells stably expressing GFP-shRNAs. The results demonstrated that the fluorescence intensity was remarkably reduced in the shDDX21-transfected PK-15 cells compared with that in the shCON-transfected cells (Figures 5A,B). Western blotting indicated that endogenous DDX21 expression was significantly decreased in the shDDX21-transfected PK-15 cells compared with that in the shCON-transfected cells (Figure 5C). Moreover, cell counting kit-8 (CCK-8) assays demonstrated that the viability or proliferation of the shDDX21-transfected cells was not affected significantly (Figure 5D, *p* > 0.05). The *DDX21*-silenced and control and DDX21-overexpressed PK-15 cells were transfected with GFP-PCV4-Cap for 24 h to investigate the subcellular localization of PCV4 Cap. Confocal imaging demonstrated that PCV4 Cap represented predominant nucleolar localization in shCON-transfected cells, and relocated to the nucleoplasm in *DDX21*-silenced cells, and restored nucleolar distribution in the DDX21-overexpressed cells (Figures 5E,F). Taken together, the results indicated that the DDX21 is required for facilitating the nucleolar distribution of PCV4 Cap.

## Discussion

In a silent state, DDX21, along with its binding ligands c-Jun, SIRT7, and WDR46, is distributed in the nucleolus (Holmstrom et al., 2008; Hirai et al., 2013; Song et al., 2017). The nucleolar localization of DDX21 is essential for its pre-rRNA processing and RNA unwinding (Holmstrom et al., 2008; Song et al., 2017). Latest reports have shown that the RNA helicase DDX21 modulates RNA virus replication through multiple pathways, such as impressing viral genome replication, and inhibiting virion assembly and release (Watanabe et al., 2009; Chen et al., 2014; Dong et al., 2016). DDX21 is a nuclear protein and binds to rRNAs and snoRNAs to promote rRNA transcription and processing (Flores-Rozas and Hurwitz, 1993; Holmstrom et al., 2008; Calo et al., 2015). Moreover, DDX21 unwinds dsRNA and RNA guanine quadruplexes (Valdez et al., 1997; McRae et al., 2017; Mcrae et al., 2018). Some studies have demonstrated that DDX21 regulates anti-virus innate immunity as well. For instance, DDX21, along with DDX1 and DHX36, can interact with TRIF to sense dsRNA (Zhang et al., 2011). DDX21 relocates to the cytoplasm from the nucleus for inducing innate immunity upon dengue virus challenge (Dong et al., 2016). Besides, the cleavage of DDX21 promotes its translocation from the nucleus to the cytoplasm and negatively modulates the IFN-β signaling pathway by attenuating the formation of the DDX1-DDX21-DHX36 complex in response to virus infection (Wu et al., 2021). Herein, we demonstrated that DDX21 relocated from the nucleus to the cytoplasm in response to PCV4 Cap expression (Figure 1), inferring that the PCV4 Cap triggered DDX21 redistribution to antagonize the host cellular innate immunity to promote circovirus replication and the precise mechanisms need further study. Thus, we proposed that the “dual” distribution of DDX21 protein may play significant roles in rRNA processing and loosening (nucleolus) and control of host innate immunity in the cytoplasm. Until now, the role of DDX21 in DNA virus infection has been reported only once (Hao et al., 2019). Thus, how DDX21 regulates porcine circovirus lifecycle remains unclear.

The NLSs of viral proteins are deemed as fundamental elements (Wurm et al., 2001; Hiscox, 2002; Salvetti and Greco, 2014). Certain viral proteins bearing NoLSs are essential for regulating host transcription, cell division, virus transcription and translation (Puviondutilleul and Christensen, 1993; Liu et al., 1997; Hiscox et al., 2001; Matthews, 2001; Wurm et al., 2001). No exact consensus sequences are found within NoLS, even though the sequences are always divided into monopartite or bipartite NoLS (Silver, 1991). The replication of circovirus genome occurs in the nucleus, and the nuclear entry of ssDNA genome needs karyophilic proteins (Heath et al., 2006). As for PCVs or beak and feather disease virus, the NoLS is required for genome replication and transcription (Cheung and Bolin, 2002; Heath et al., 2006). The N-terminus of PCV2 Cap can interact with gC1qR on the nuclear membrane for regulating DNA binding (Fotso et al., 2016). This indicates that the NoLS of PCV4 Cap can also facilitate DNA binding for modulating virus replication.

Our results showed that PCV4 Cap binds directly to nucleolar protein DDX21, and the amino acid residues 1–37 at the N-terminus of PCV4 Cap are required for relatedness to DDX21 (Figures 2, 3). Additionally, we verified that ^763^GSRSNRFQNK^772^ of DDX21-CTD is crucial for interaction with the PCV4 Cap NoLS (Figure 4). As the nucleolar localization signal (NoLS) at the N-terminus of PCV4 Cap serves as an DDX21 binding site, we hypothesize that DDX21 facilitates intracellular nucleolar trafficking of PCV4 Cap (Figure 5). Previous reports showed that the C-terminus of DDX21 bound to c-Jun, and the depletion of c-Jun facilitates DDX21 translocation from the nucleolus to the nucleoplasm (Holmstrom et al., 2008). Therefore, it is possible that the C-terminus deletion of DDX21 abrogates the interaction with its binding ligand and thus alters its nucleolar distribution. The DDX21 targets the PCV4 Cap to the nucleolus via binding to its NoLS thereby facilitating assembly of viral particles, hence it contributes to virus replication inside the nucleus. PCV Cap might gain entry into the nucleolus to facilitate viral genome replication and transcription, or to hijack the S phase cell cycle and synthesize host proteins at the early phase of infection (Finsterbusch et al., 2005). It will be worth demonstrating if PCV4 Cap binding to other cellular factors to regulate viral transcription and genome replication.

Herein, we demonstrated that the PCV4 Cap induced translocation of DDX21 to the nucleolus from the cytoplasm. In addition, we verified that the PCV4 Cap NoLS bound to DDX21. Moreover, the NoLS of the PCV4 Cap and ^763^GSRSNRFQNK^772^ residues at the CTD of DDX21 were essential for the PCV4 Cap/DDX21 interaction. Furthermore, the PCV4 Cap NoLS exploited DDX21 to facilitate its nucleolar localization. Collectively, our findings for the first time demonstrated that DDX21 binds directly to the PCV4 Cap NoLS and is responsible for its nucleolar localization, thereby contributing to discovering novel potential targets for prevention and control of PCV4 infection.

## Materials and Methods

### Cells

PK-15 cells were cultured in minimal essential medium (MEM) (Gibco, Carlsbad, CA, United States) supplemented with 10% fetal bovine serum (FBS) (Gibco). HEK293T cells (CRL-11268; ATCC, Manassas, VA, United States) were maintained in Dulbecco’s modified Eagle medium (DMEM) (Gibco) as described elsewhere (Zhou et al., 2020b,^2021^).

### Antibodies and Reagents

Mouse monoclonal antibodies (mAbs) against GST (M0807-1), histone H3 (R1105-1), and β-actin (M1210-2), as well as rabbit polyclonal antibodies (pAbs) against Myc (R1208-1), Flag (0912-1), β-tubulin (0807-2), and GFP (SR48-02) were purchased from Huaan Biological Technology (Hangzhou, China). Mouse anti-Flag (F1804) and anti-Myc (05–419) mAbs were obtained from Sigma-Aldrich (St. Louis, MO, United States). Rabbit mAb against DDX21 (ab182156) was obtained from Abcam (Cambridge, MA). Anti-Flag affinity resin (A2220) for immunoprecipitation was purchased from Sigma-Aldrich. NP-40 cell lysis buffer (50 mM Tris [pH 7.4], 150 mM NaCl, 1% NP-40) was obtained from Beyotime (P0013F; Shanghai, China). Horseradish peroxidase (HRP)-labeled goat anti-mouse and anti-rabbit IgG or fluorescein isothiocyanate (FITC)-labeled goat anti-mouse IgG were purchased from KPL (Milford, MA, United States). Alexa Fluor 546-conjugated donkey anti-rabbit IgG was obtained from Invitrogen (United States).

### Plasmid Construction and Cell Transfection

Full-length and truncated PCV4 *Cap* DNA fragments were amplified by polymerase chain reaction (PCR) from synthetic PCV4 genomic DNA (accession no. MK986820.1) (Zhang et al., 2019), and inserted into the multiple cloning site of vectors pcDNA3.0 (Invitrogen), pCMV-Myc-N, pCMV-Flag-N, pEGFP-C3, or pCMV-Flag-gst-N (Clontech, Palo Alto, CA, United States) to obtain plasmids pcDNA3.0-PCV4-Cap, Myc-PCV4-Cap, Flag-PCV4-Cap, GFP-PCV4-Cap-WT-(1–228 aa), GFP-PCV4-Cap-M1-(38–228 aa), GFP-PCV4-Cap-M2-(1–37 aa), Flag-gst-PCV4-Cap-WT-(1–228 aa), Flag-gst-PCV4-Cap-M1-(38–228 aa), or Flag-gst-PCV4-Cap-M2-(1–37 aa). The nucleotide fragment *sumo-PCV4-Cap* was synthesized and cloned into the vector pET-28a (Invitrogen, Carlsbad, CA, United States) by Sangon Biotechnology (Shanghai, China). The NoLSs within capsid protein of different porcine circoviruses genotypes are listed in Table 1. The *DDX21* (accession no. KX396051.1) and its truncated *DDX21* variants amplified from PK-15 cells were cloned into vectors pCMV-Flag-N, pGEX-4T-1 (GE Healthcare Biosciences, Piscataway, NJ, United States), or pEGFP-C3 to obtain plasmids Flag-DDX21, GST-DDX21, GFP-DDX21-WT-(1–784 aa), GFP-DDX21-M1-(1–217 aa), GFP-DDX21-M2-(218–581 aa), GFP-DDX21-M3-(582–784 aa), GFP-DDX21-M4-(1–581 aa), GFP-DDX21-M5-(218–784 aa), GFP-DDX21-M6-(1–217 aa + 582–784 aa), GFP-DDX21-M7-(582–772 aa), GFP-DDX21-M8-(582–762 aa), or GFP-DDX21-M9-(763–772 aa). The detailed procedures for plasmids construction were conducted as described elsewhere (Zhou et al., 2020a,b). The primers adopted are summarized in Table 2. PK-15 or HEK293T cells grown onto plates or glass coverslips up to 70–90% confluency were transfected or co-transfected with 4.0 μg of the, respectively, indicated plasmids. The jetPRIME transfection reagent (Polyplus Transfection, New York, NY, United States) was used for PK-15 cell transfection, and the ExFect transfection reagent (T101-01/02; Vazyme Biotechnology, Nanjing, China) was used for HEK293T cell transfection as described in the manufactures’ protocols.

**TABLE 1:** The nucleolar localization signals (NoLSs) of Cap protein from different porcine circovirus genotypes.

Circoviruses	Accession no.	Sequences of NoLSs
PCV1	AY193712.1	MTWPRRRYRRRRTRPRSHLGNILRRRPYLAHPAFRNRYRWRRK
PCV2	AY188355.1	MTYPRRRYRRRRHRPRSHLGQILRRRPWLVHPRHRYRWRRK
PCV3	KT869077.1	MRHRAIFRRRPRPRRRRRHRRRYARRRLFIRRPT
PCV4	MK986820.1	MPIRSRYSRRRRNRRNQRRRGLWPRANRRRYRWRRKN

**TABLE 2:** List of primers adopted in the study.

Gene product	Sense primer (5′–3′)	Antisense primer (5′–3′)
PCV4 Cap (1–228 aa)	ATGCCAATCAGATCTAGGTACA	TTATCCCTGTTTGGGGTAGTTAACA
PCV4 Cap (38–228 aa)	CATGCGCGCTTCATGAGGGA	TTATCCCTGTTTGGGGTAGTTAACA
PCV4 Cap (1–37 aa)	ATGCCAATCAGATCTAGGTACA	GTTCTTCCTTCTCCACCGGTATCTC
DDX21 (1–784 aa)	ATGCCGGGGAAACTTCGTAGT	TTACTGTCCAAACGCTTTGCT
DDX21 (1–217 aa)	ATGCCGGGGAAACTTCGTAGT	CGTCTTTGCTTGTATGGGAAACA
DDX21 (218–581 aa)	TTTCACCATGTCTATAGCGGGAA	GATGGCATCTTTACTAGAAGCTTTT
DDX21 (582–784 aa)	AGGCTTTTGGATTCTGTGCCT	TTACTGTCCAAACGCTTTGCT
DDX21 (1–581 aa)	ATGCCGGGGAAACTTCGTAGT	GATGGCATCTTTACTAGAAGCTTTT
DDX21 (218–784 aa)	TTTCACCATGTCTATAGCGGGAA	TTACTGTCCAAACGCTTTGCT
DDX21 (1–217 aa + 582–784 aa)	ATGCCGGGGAAACTTCGTAGT	AGGCACAGAATCCAAAAGCCTCGTCTTTGCTTGTAT
	ATACAAGCAAAGACGAGGCTTTTGGATTCTGTGCCT	TTACTGTCCAAACGCTTTGCT
	ATGCCGGGGAAACTTCGTAGT	TTACTGTCCAAACGCTTTGCT
DDX21 (582–772 aa)	AGGCTTTTGGATTCTGTGCCT	TTTGTTTTGGAATCTGTTGCTT
DDX21 (582–762 aa)	AGGCTTTTGGATTCTGTGCCT	ACCTCCTGATCGCTGTCCCCTGAA
DDX21 (763–772 aa)	TCGAGGGTAGCAGAAGCAACAGATTCCAAAACAAATAAG	AATTCTTATTTGTTTTGGAATCTGTTGCTTCTGCTACCC

### Confocal Microscopy

PK-15 cells were transfected with plasmids pcDNA3.0-PCV4-Cap or mCherry-PCV4-Cap. The cells were washed with PBS and fixed with 4% paraformaldehyde for 10 min and then permeabilized with 0.1% Triton-X 100 for 10 min at room temperature. Mouse anti-PCV4 Cap pAb and rabbit anti-DDX21 mAb were used as primary antibodies, and fluorescein isothiocyanate (FITC)-conjugated goat anti-mouse IgG and Alexa Fluor 546-conjugated donkey anti-rabbit IgG were used as secondary antibodies. Cellular nuclei were stained with 10 μg/ml 4′, 6′-diamidino-2-phenylindole (DAPI; 10236276001; Roche, Mannheim, Germany) to obtain images using a Nikon Al confocal microscope.

### Dodecyl Sulfate-Polyacrylamide Gel Electrophoresis and Western Blotting

Cell lysate extracts prepared in lysis buffer after transfection were separated by standard sodium dodecyl sulfate-polyacrylamide gel electrophoresis (SDS-PAGE) and transferred to nitrocellulose membranes (GE Healthcare) followed by being blocked in phosphate-buffered saline (PBS) containing 5% skimmed milk powder and 0.05% Tween 20. The membranes were then incubated with primary antibodies overnight at 4°C followed by incubation with HRP-labeled secondary antibodies at room temperature for 1.0 h. The membranes were then incubated with enhanced chemiluminescence reagent (34096; Thermo Scientific) and the immunoreactive protein bands were visualized using AI800 Images (GE Healthcare).

### Co-immunoprecipitation and Glutathione S-Transferase Pull-Down Assays

For co-immunoprecipitation (Co-IP) assays, HEK293T cells transfected with the indicated plasmids for 48 h were lysed in NP-40 cell lysis buffer and centrifugated at 12,000 × *g* for 10 min. The supernatants were treated with protein A/G plus agarose (sc-2002; Santa Cruz Biotechnology, CA, United States) for 1.0 h at 4°C and then immunoprecipitated using anti-Flag beads. The beads were washed with NP-40 buffer and then revolved by the standard SDS-PAGE. For GST pull-down assays, the expression of His-sumo-PCV4-Cap, GST, or GST-DDX21 in *Escherichia coli* BL21 cells was induced using 0.2 mM isopropyl-β-D-thiogalactopyranoside (Amersham) for 16 h at 16°C. GST and GST-DDX21 were obtained through incubation with Pierce glutathione agarose beads (21516; Thermo, Rockford, IL, United States) and were eluted with 1.0 ml of cold 1 × PBS per 10 mg of reduced glutathione. His-sumo-PCV4-Cap was purified using NTA agarose affinity resin (30210; QIAGEN, Hilden, Germany) and eluted using an imidazole concentration gradient and used as the prey protein. Equal amount of purified GST or GST-DDX21 proteins which were immobilized on glutathione agarose beads, were incubated with the corresponding prey proteins at 4°C for 6.0 h. The precipitated proteins were washed with PBS, subjected to SDS-PAGE and western blotting using mouse mAbs against GST and His. The procedures for Co-IP and the unconventional GST pull-down assays were conducted as described elsewhere (Zhou et al., 2020a,b).

### Nuclear and Cytoplasmic Extraction

Isolation of nuclear and cytoplasmic components was performed as stated in our previous study with moderate modifications (Zhou et al., 2020b). According to the protocol, pcDNA3.0-PCV4-Cap-transfected PK-15 cells were lysed using 0.1% NP-40 lysis buffer supplemented with 1.0 mM phenylmethylsulfonyl fluoride (PMSF) (ST506; Beyotime) at 4°C for 5.0 min. After centrifugation at 1,000 × *g* for 5.0 min, the supernatants of the samples were collected as the cytoplasmic fractions, while the precipitate was lysed with strong lysis buffer and used as the nuclear fractions. Western blotting analysis was performed using mouse pAb anti-PCV4 Cap, mouse mAb against histone H3, and rabbit pAb against β-tubulin.

### *DDX21* Knockdown by Lentivirus-Mediated RNA Interference

*DDX21* knockdown was performed as previously described (Zhou et al., 2020b) with slight modifications. Briefly, three pairs of shRNA targeting porcine *DDX21* (shDDX21-1, -2, -3) were designed using siRNA design software and cloned into the lentivector pGreenPuro shRNA vector (SI505A 1; System Biosciences, Palo Alto, CA, United States). After transfection and selection, an effective shDDX21 (targeting sequence: GCAGAGACTTCAGTGACATCA) and shCON, which was developed previously (Zhou et al., 2020b), were co-transfected using a ViraPower Lentiviral Packaging Mix (Thermo) into HEK293T cells for 48 h. PK-15 cells were then infected with the resultant lentiviruses, cultured for 24 h, and selected using puromycin (5 μg/ml) (A1113803; Invitrogen) for a week to obtain *DDX21*-knockdown cells. Finally, western blotting analysis was performed using rabbit mAb against DDX21 and mouse mAb against β-actin. Cell viability was determined using a cell counting kit-8 (CCK-8) (C0037; Beyotime) assay according to the manufacturer’s protocol.

### Statistical Analysis

All results are presented as means ± standard deviations (SD). Statistical analysis was performed using Student’s *t*-test. *p*-values of < 0.05 were considered significant.

## Data Availability Statement

The original contributions presented in the study are included in the article/supplementary material, further inquiries can be directed to the corresponding author/s.

## Author Contributions

JZ conceived, designed, performed the experiments, interpreted the data, and wrote the manuscript. JZ performed the experiments with assistance and advice from YW, YQ, YxW, XY, CL, YS, XF, and LH. JL revised the manuscript. All authors have read and approved the submission.

## Conflict of Interest

The authors declare that the research was conducted in the absence of any commercial or financial relationships that could be construed as a potential conflict of interest.

## Publisher’s Note

All claims expressed in this article are solely those of the authors and do not necessarily represent those of their affiliated organizations, or those of the publisher, the editors and the reviewers. Any product that may be evaluated in this article, or claim that may be made by its manufacturer, is not guaranteed or endorsed by the publisher.

## Footnotes

1
http://www.compbio.dundee.ac.uk/www-nod/index.jsp

2
http://nls-mapper.iab.keio.ac.jp/cgi-bin/NLS_Mapper_form.cgi
